# Comparison of a minimally invasive osteosynthesis technique with open surgical technique for transverse patellar fractures

**DOI:** 10.1097/MD.0000000000042397

**Published:** 2025-06-06

**Authors:** Xiang-Yu Ma, Dong Cui, Jia-Lin Sun, Hong Yuan, Bing Liu, Da-Peng Zhou, Hai-Long Yu, Tian-Yu Han

**Affiliations:** a Department of Orthopedics, General Hospital of Northern Theatre Command, Shenyang, Liaoning Province, China; b Department of Cardiology, No.967 Hospital of PLA Joint Logistics Support Force, Dalian, Liaoning Province, China.

**Keywords:** minimally invasive osteosynthesis, modified carpenter technique, open surgery, transverse patellar fractures

## Abstract

This retrospective study aimed to compare the safety and efficacy of a new minimally invasive osteosynthesis technique with those of conventional open surgery for transverse patellar fractures. Between January 2016 and December 2022, a total of 138 patients with transverse patellar fractures who underwent osteosynthesis with either minimally invasive osteosynthesis technique (MIOT) or open reduction and internal fixation (ORIF) were enrolled and retrospectively analyzed. The outcomes were assessed for 67 patients in the MIOT group (mean age: 46.2 ± 15.8 years old, mean follow-up: 26.4 ± 5.1 months) and 51 patients in the ORIF group (mean age: 43.7 ± 13.4 years old, mean follow-up: 25.1 ± 4.8 months). Clinical outcomes, including surgical time, blood loss, bony union time, final range of motion involving knee extension and flexion, Bostman score, visual analogue scale (VAS), and complications, were measured over a minimum follow-up period of 24 months. The surgical time in the MIOT group was shorter than that in the ORIF group (*P* = .001). The blood loss in the MIOT group was significantly less than that in the ORIF group (*P* < .0001). At the 2-year follow-up, all fractures had healed. The mean union time in the MIOT group was shorter than that in the ORIF group (*P* = .002). The MIOT group also exhibited significantly better flexion (*P* = .001) and a higher Bostman score (*P* = .0065), compared with the ORIF group. The mean VAS was significantly lower in the MIOT group than that in the ORIF group (*P* < .0001). The MIOT group had a lower complication rate, including delayed wound healing and implant irritation, as well as an overall lower complication rate. The MIOT method proved to be a reproducibly reliable approach, offering lower surgical trauma, improved functional outcomes, and a lower incidence of complications compared with the conventional open surgical technique for transverse patellar fractures. It may be a prudent choice for treating transverse patellar fractures.

## 1. Introduction

Patella fractures comprise 0.5% to 1.5% of all skeletal fractures. Transverse fractures account for more than 70% of patella fractures, which cause functional disability of the knee extensor mechanism.^[1]^ Surgical intervention is indicated for displaced fractures with a step‑like discontinuity of the articular surface exceeding 2 mm or a fracture gap exceeding 3 mm.^[2]^ Open reduction and internal fixation (ORIF) utilizing tension band wiring stand as the prevailing approach for transverse patellar fractures, yielding satisfactory clinical outcomes.^[3,4]^ Carpenter et al^[5]^ firstly introduced figure-eight wiring through parallel cannulated screws via open surgery for transverse patellar fractures, demonstrating its superior fixation compared to other methods.^[6]^ However, the open approach poses challenges, causing significant soft tissue and skin damage, potential impact on patellar blood supply,^[7]^ and associated complications such as delayed wound healing, infection, implant irritation, fixation failure and revision. Furthermore, postoperative incision adhesion which affects knee joint motion range is a common postoperative event.^[8,9]^

Theoretically, 2-part transverse patellar fractures are more amenable for anatomic reduction through closed manipulation for percutaneous osteosynthesis, which is increasingly favored by clinicians nowadays.^[10]^ The minimally invasive osteosynthesis technique (MIOT) is an indirect or limited open reduction technique involving small incisions, percutaneous reductions and implant fixations. It offers potential advantages including reduced postoperative wound complications, decreased delayed operation risks, and enhanced early rehabilitation.^[11]^ By refining surgical procedures, MIOT achieves stability through percutaneous fixations involving K-wires and cannulated screws.^[12–14]^ Some studies suggested arthroscopy assistance for better evaluation of articular congruity with a magnified visual field,^[11,15]^ albeit at the cost of complexity and expense.

Despite the potential benefits, the adoption of minimally invasive percutaneous fixation for patellar fractures remains limited due to insufficient research on its merits, complications, and the scarcity of comparisons with traditional open tension band wiring. In recent years, we implemented a minimally invasive technique using figure-eight wiring through 2 parallel cannulated screws, akin to Carpenter method, for transverse patellar fractures. In this technique, we implanted 2 parallel cannulated screws, through which 2 separate wires were passed and twisted in figure-eight for rigid fixation.

This retrospective non-randomized study aimed to compare the efficacies of MIOT with ORIF in management of transverse patellar fractures in adult patients, evaluating whether MIOT demonstrated superior clinical outcomes through detailed statistical analyses.

## 2. Materials and methods

### 2.1. Patient demographics

The present research was a retrospective, observational study approved by the Ethics Committee of General Hospital of Northern Theater Command (Y (2024) 373). The study was conducted according to the principles of the Declaration of Helsinki strictly. Written informed consents were obtained from all of the enrolled patients. Between January 2016 and December 2022, a total of 156 patients with patellar transverse fractures treated in our hospital were retrospectively recruited.

Inclusion criteria were: simple transverse pattern of fracture (type 34-C1 according to AO/OTA); treated with either percutaneous osteosynthesis using the MIOT method or conventional ORIF technique; acute fractures with articular displacement ≥ 2 mm; unilateral patellar fracture. Exclusion criteria were: open fractures; pathological skeletal disease or serious underlying diseases; (3) history of previous knee surgery; A follow-up of <24 months. Of the 156 patients, 8 fractures in the MIOT group and 10 in the ORIF group were lost to follow-up after 1 year, leaving 138 patients as study subjects, categorized into MIOT (n = 67) and ORIF (n = 71) groups based on the internal fixation method. The patient grouping criteria was based on surgeon preferences but not fracture severity to mitigate selection bias. The demographic characteristics of enrolled patients were presented in Table 1.

**Table 1:** Patient characteristics.

Charateristics	MIOT group (67)	ORIF group (71)	*χ*^2^/*t*-value	*P*-value
Gender				
Male	43	44	0.072	.788
Female	24	27		
Age	46.2 ± 15.8	43.7 ± 13.4	1.004	.317
Fractured side				
Left	36	34	0.471	.493
Right	31	37		
Mechanism of injury				
Traffic accident	23	20	1.002	.801
High-energy fall	28	35		
Sport injury	10	9		
Others	6	7		
AO/OTA type				
Transverse, middle (45-C1.1)	57	62	0.147	.702
Transverse, distal (45-C1.3)	10	9		

MIOT = minimally invasive osteosynthesis technique, ORIF = open reduction and internal fixation.

**Figure 1.**
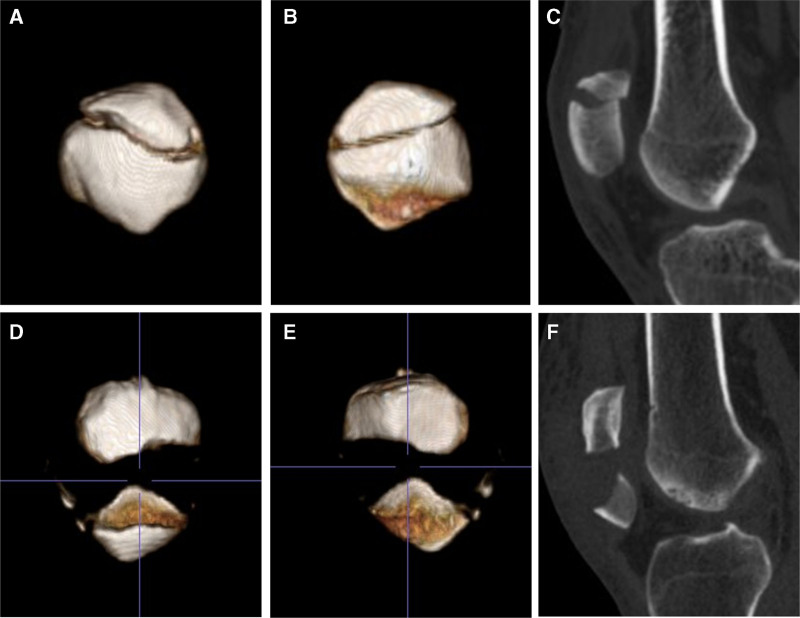
The preoperative CT images of the typical patients in both groups. (A) and (B) 3D-CT images of a 40-year-old male (typical Patient 1) with a mildly displaced transverse patellar fracture; (C) the sagittal view of the fracture; (D) and (E) 3D-CT images of a 40-year-old male (typical Patient 2) with an obviously displaced transverse patellar fracture; (F) the sagittal view of the fracture. CT = computed tomography.

The 3D-computed tomography scans were taken preoperatively to evaluate all the fractures (Fig. 1). All the surgical procedures were performed by the same surgical and anesthesiology team, under spinal or general anesthesia. The patient was placed in supine position with slight flexion of knee joint and a tourniquet was applied to minimize the blood loss.

### 2.2. MIOT technique

**Figure 2.**
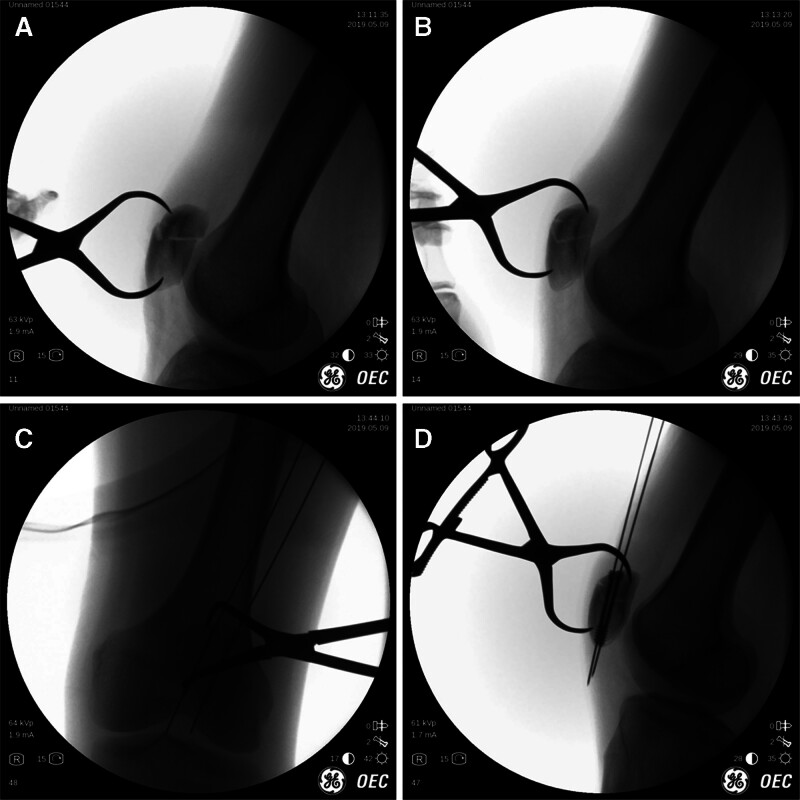
Percutaneous reduction and cannulated screw fixation assessed with C-arm. (A) Application of a reduction clamp across fragments percutaneously, (B) satisfactory reduction achieved with the clamp, (C) anteroposterior, and (D) lateral X-ray films of cannulated screw fixation through the guide wires.

In the patients undergoing MIOT surgical procedure, the upper pole of the proximal fragment and the lower pole of the distal fragment were identified by palpation and confirmed with fluoroscopy. A pointed reduction clamp was percutaneously applied at the far end of each fragment (Fig. 2A). The fracture was temporarily reduced by manually compressing the reduction clamp with the knee joint in full extension, and verified anteroposteriorly and laterally with fluoroscopy (Fig. 2B). If joint congruity was not achieved, further manipulation was performed until satisfactory congruity was attained.

The knee was then flexed to 15° to facilitate the insertion of guide wires. Two guide wires which were separated by 1.5 to 2 cm were inserted from the proximal end of the patella through 2 small stab incisions under fluoroscopy guidance. The guide wires were advanced until they could be felt beneath the skin at the distal pole of patella, where 2 additional stab incisions were made. The lengths of screws were slightly shorter than the initial measurement to prevent penetration of the lower pole by screw tips, minimizing stress concentration and potential breaking in the tension band. After sequential drilling through the guide wire, 2 partially-threaded 4.0-mm cannulated screws were inserted (Fig. 2C and D).

**Figure 3.**
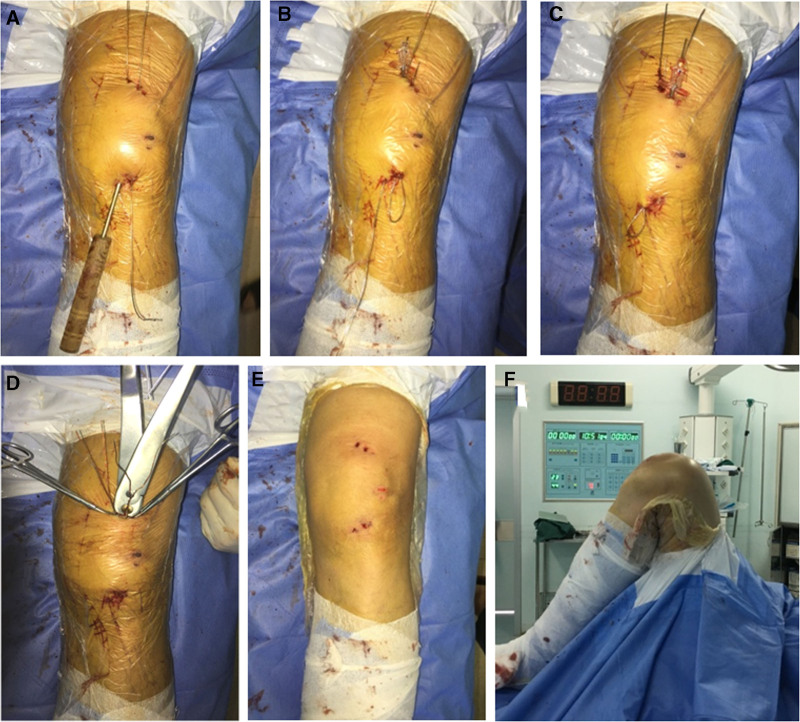
Figure-eight wiring through the parallel cannulated screws on the surgical field. (A) Insertion of the 2 wires retrogradely through the cannulated screws, (B) and (C) percutaneous passage of the 2 wires through the guided cannula, (D) cutting of the residual knots after tightening the 2 twisted wires, (E) the surgical incision with the MIOT, and (F) introoperative evaluation of flexion in patient 1.

**Figure 4.**
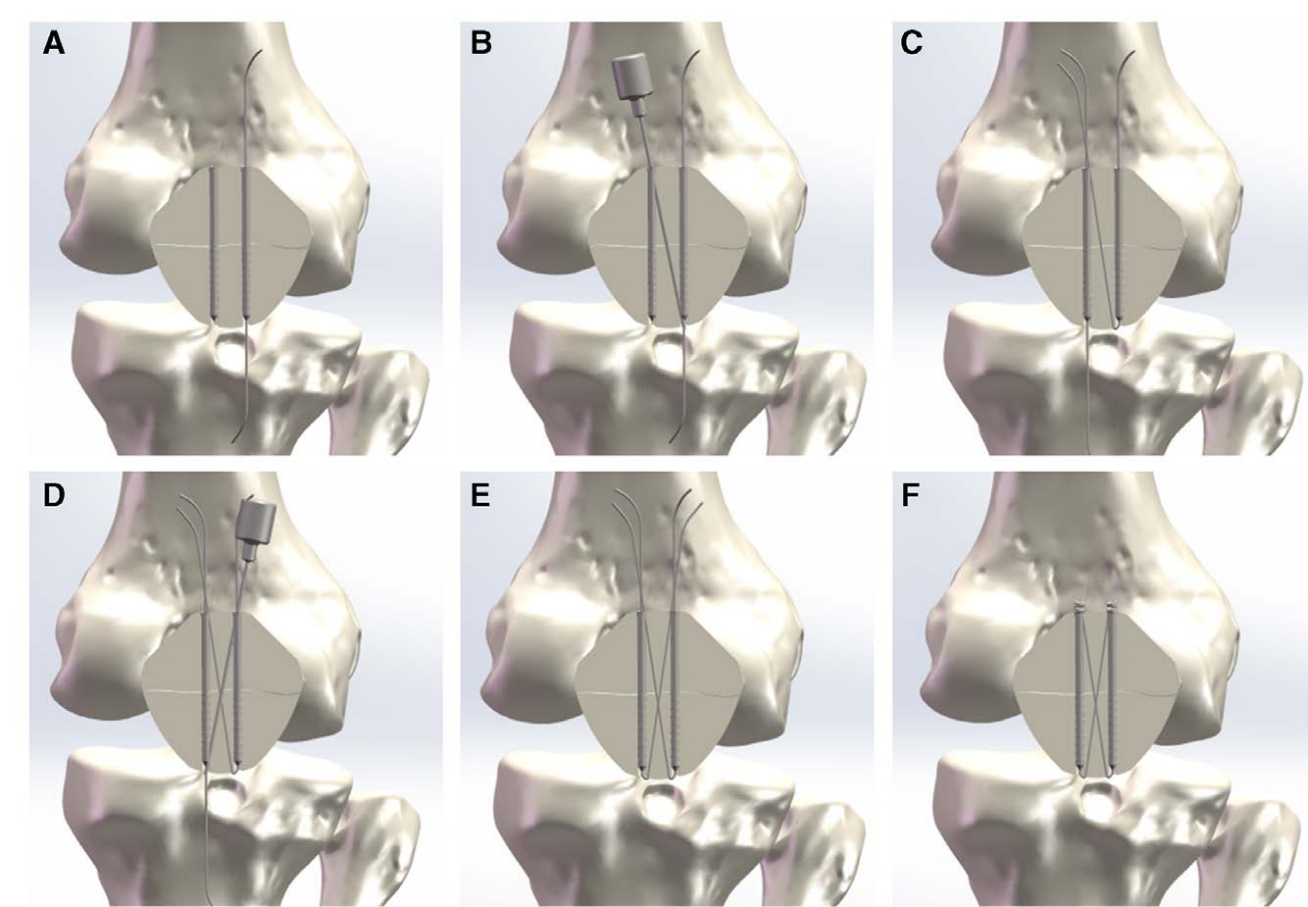
The technical drawing of the minimally invasive surgical technique. (A) The first wire retrogradely passing through the lateral cannulated screw after insertion of the cannulated screws. (B) Guided suction tip insertion from superomedial to inferolateral for percutaneous passage of the first wire. (C) The second wire passing through the medial cannulated screw retrogradely meeting the first wire in the superomedial incision. (D) Guided suction tip insertion from superolateral to inferomedial for percutaneous passage of the second wire. (E) Each end of the 2 wires emerging out of the screw heads in superolateral and superomedial. (F) Cutting of the residual knots close to the screw head after tightening the 2 twisted wires.

After the removal of the guide wires and reduction clamps, the 2 wires with diameter of 18 gauge (1.2 mm) were retrogradely passed through the cannulated screws, with wire ends exiting the proximal stab incisions (Figs. 3A and 4A). A suction tip served as a cannula, passed subcutaneously from the proximal stab incision of the medial screw to the distal stab incision of the lateral screw (Fig. 4B). The head of the lateral wire was bent and guided within the tip back toward the proximal stab incision of the medial screw (Figs. 3B and 4C). The same procedure was repeated for the medial wire, directed to the proximal stab incision of the lateral screw (Figs. 3C and 4D). The wire ends at the proximal stab incisions were tightened (Fig. 4E) and knotted simultaneously until touching the screw heads. The residual knots were cut off (Figs. 3D and 4F). Finally the surgical wounds were closed with cosmetic sutures, with knot ends band-fixed under the incisions (Fig. 3E). The reduction and tension of fixation were checked to 150° of flexion during the operation (Fig. 3F).

### 2.3. ORIF technique

**Figure 5.**
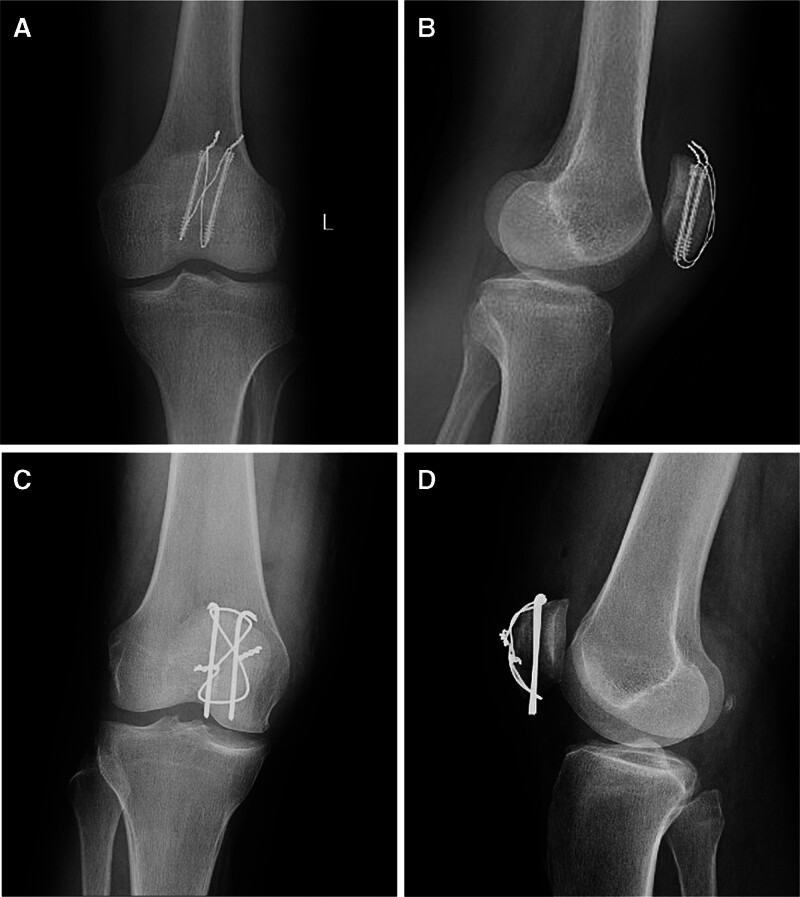
The 2-d postoperative radiographs of fracture reductions and implant positions. (A) Posteroanterior and (B) lateral X-rays of Patient 1 at 2 d post-operation with the MIOT technique; (C) posteroanterior and (D) lateral X-rays of patient 2 at 2 d post-operation with the open surgical technique.

In the patients undergoing ORIF surgical procedure, a longitudinal midline incision with the length of 8 to 12 cm was made on the anterior aspect of knee. Subsequently, the subcutaneous tissue was fully separated to expose the fracture site. The fracture fragments were anatomically reduced using a reduction clamp and fixed with 2 K-wires and a tension band in an “8” pattern under clear view. Finally, the surgical incision was closed in layers over a negative suction drain, which was removed 2 days post-operation. The total operative time was calculated from incision to skin closure for all patients. Radiological evaluations, including posteroanterior and lateral X-rays of knee joint, were performed at 2 days post-operation (Fig. 5).

### 2.4. Rehabilitation

No splint or brace was used for all the patients in both groups. Unrestricted passive ROM commenced postoperatively as much pain as the patient could tolerate. On postoperative day 1, partial weight-bearing was allowed in association with crutches and knee brace locked in extension. Upon achieving satisfactory quadriceps control, the brace was removed. The full weight-bearing initiated after 4 to 6 weeks.

### 2.5. Postoperative follow‑up and clinical/radiological assessment

All the patients were followed biweekly within the first month, then monthly until 6 months, and every 6 months thereafter until the last definitive follow-up. The bony union time of fracture was documented postoperatively on standard anteroposterior and lateral X-ray radiographs, which were read by 2 well-trained orthopedic surgeons independently. The bony union was defined as the loss of the fracture line and the presence of bony trabecular continuity as depicted before.^[16]^

The range of motion (ROM) of knee joint at the 2-year follow-up was measured in degrees by a goniometry. The knee joint function was evaluated using the commonly used Bostman Score System as depicted before.^[17]^ The total score was stratified as follows: 28 to 30, excellent; 20 to 27, good; <20, unsatisfactory. At 8 weeks, patients’ subjective pain perception was quantified using the visual analog scale (VAS) ranging from 0 (no pain) to 10 (the most intense pain).

### 2.6. Statistical analysis

All statistical analyses were performed using SPSS version 24.0 software (IBM Corp., Chicago). For quantitative variables, the assumption of normality was verified by the Shapiro–Wilk test. In this study, all the continuous variables were normally distributed and presented as means ± standard deviations and analyzed using a Student *t* test. Categorical variables were compared via χ^2^ test to indicate the group differences.

## 3. Results

As shown in Table 1, no statistically significant differences were identified in gender, age distribution, fractured side, mechanism of injury, or type of fractures. The surgical and functional outcomes were presented in Table 2. None of the MIOT operations were abandoned and converted to open surgery. The surgical time in the MIOT group (63.5 ± 10.3 minutes) was significantly shorter compared to the ORIF group (70.1 ± 12.6 minutes, *P* = .001). The MIOT group exhibited significantly less blood loss (5.5 ± 1.3 mL) compared to the ORIF group (76.5 ± 18.7 mL, *P* < .0001).

**Table 2:** Surgical and functional outcomes.

Variables	MIOT group (67)	ORIF group (71)	*χ*^2^/*t*-value	*P*-value
Surgical time (min)	63.5 ± 10.3	70.1 ± 12.6	3.358	.001
Blood loss (mL)	5.5 ± 1.3	76.5 ± 18.7	31.92	<.0001
follow up (mo)	26.4 ± 5.1	25.1 ± 4.8	1.543	.125
Union time (wk)	10.2 ± 1.9	11.3 ± 2.2	3.135	.002
Extension (°)	-0.8 ± 3.0	-1.1 ± 3.2	0.567	.571
Flexion (°)	143.8 ± 15.1	135.7 ± 12.8	3.406	.001
Bostman score	28.4 ± 3.1	27.1 ± 2.4	2.763	.0065
VAS	1.2 ± 0.3	4.3 ± 1.0	24.36	<.0001

MIOT = minimally invasive osteosynthesis technique, ORIF = open reduction and internal fixation, VAS = visual analogue scale.

**Figure 6.**
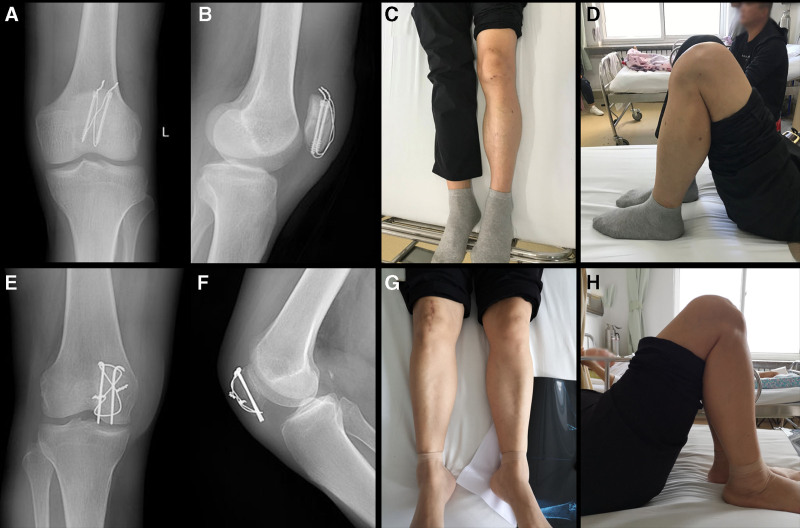
Clinical evaluation at 1-year follow-up. (A) Posteroanterior and (B) lateral X-rays of Patient 1 at 12 mo after surgery showing bone healing, (C) appearance of the minimally invasive surgical scar in the extension position, (D) evaluation of knee joint flexion. (E) Posteroanterior and (F) lateral X-rays of patient 2 at 12 mo after surgery showing bone healing, (G) appearance of the large surgical scar in the extension position, (H) evaluation of knee joint flexion.

All patients completed ≥ 2 years of follow-up, and all fractures healed (Fig. 6A, B, E, and F). The mean union time of the MIOT group was shorter (10.2 ± 1.9 weeks) than that of the ORIF group (11.3 ± 2.2 weeks, *P* = .002). At the last follow-up, the MIOT group showed significantly better flexion (143.8 ± 15.1°) compared to the ORIF group (135.7 ± 12.8°, *P* = .001, Fig. 6C, D, G, and H). The Bostman score was higher in the MIOT group (28.4 ± 3.1) than the ORIF group (27.1 ± 2.4, *P* = .0065). The mean VAS was significantly lower in the MIOT group (1.2 ± 0.3) compared to the ORIF group (4.3 ± 1.0, *P* < .0001).

All the complications in the follow-up period of both groups were shown in Table 3. The overall frequency of complications was 26.8% in the ORIF group, which was significantly higher than 6.0% in the MIOT group (*P* < .0001). During the follow-up period, no defects of delayed wound healing (defined as wound healing more than 14 days), infection (including wound redness and purulent exudation^[14]^) or implant migration were detected in the MIOT group. The ORIF group showed delayed wound healing (6 cases) and wound infection (1 case), managed with adjusted antibiotics. Implant migration occurred in 2 ORIF cases, resolved with external plaster. Implant irritation occurred in 1 patient in the MIOT group and 8 patients in the ORIF group, and 3 in the ORIF group had to undergo removal of the symptomatic hardware at 9 months after surgery. Broken wires were reported in 1 MIOT and 2 ORIF cases. The ORIF group exhibited more cases of delayed wound healing and implant irritation than the MIOT group (*P* = .015 and 0.021, respectively).

**Table 3:** Postoperative complications.

Complications	MIOT group (67)	ORIF group (71)	*χ*^2^/*t*-value	*P*-value
Delayed wound healing	0	6	5.876	.015
Infection	0	1	0.944	.331
Implant migration	0	2	1.901	.168
Implant irritation	1	8	5.364	.021
Broken wires	1	2	0.000	1.000
Total	2	19	15.103	<.0001

MIOT = minimally invasive osteosynthesis technique, ORIF = open reduction and internal fixation.

## 4. Discussion

Our study results highlighted the efficacy of the MIOT technique in reducing surgical time, blood loss, and complications while enhancing osteosynthesis and functional outcomes compared to open surgical techniques. This retrospective comparative study stood as one of the largest research in assessing minimally invasive techniques for managing patellar fractures to our knowledge. The primary goals of surgical treatment for transverse patellar fractures involve achieving anatomical reduction of the articular surface and restoring the knee extensor mechanism, thereby establishing stable fixation for early ROM.^[3]^ In the contemporary landscape, minimally invasive techniques are gaining prominence across various fracture types. Several studies, including those focused on patellar fractures, have underscored the safety and effectiveness of these minimally invasive approaches.^[11,18–20]^ Some investigations have explored the utility of arthroscopy in aiding reduction and percutaneous fixation of displaced patellar fractures.^[21,22]^ However, the technique was technically demanding and not necessary for the simple fractures.

In pursuit of a more minimally invasive objective, our study introduced a novel technique: figure-eight wiring through 2 parallel cannulated screws for treating simple patellar fractures. This percutaneous fixation method represented a modification of Carpenter approach, where Carpenter used a single wire passing through 2 cannulated screws. In contrast, our approach employed 2 separate wires, facilitating easier passage through the cannulated screws and allowing the twisting of 2 wires for additional strength. This modification didn’t introduce extra incisions or increase incision length. The incisions’ length remained almost under 1 cm, involving 2 bone tunnels and 2 subcutaneous tunnels, steering clear of extensive soft tissue dissections. All the equipment and instruments required for this technique were commonly available for orthropaedic surgeons. So this technique was both feasible and reproducible with a short leraning curve.

Surgical time in the MIOT group markedly outpaced that in the ORIF group. While the MIOT technique demanded more fluoroscopies for percutaneous closed reduction of the patella articular surface, the minimally invasive approach expedited wound closure, thereby significantly reducing blood loss. In the realm of minimally invasive techniques, percutaneous closed reduction emerged not only as a critical step but also as a technically demanding skill for surgeons. As a testament to this, arthroscopy has been proposed to assist in fracture reduction.^[11]^

Throughout the follow-up period, the MIOT group exhibited a shorter bone union time, largely attributed to the rigid fixation provided by cannulated screws with figure-eight wiring, acting as a tension band for additional compression at the fracture site. A previous biomechanical analysis by Carpenter et al demonstrated that figure-eight wiring through paired cannulated screws offered superior mechanical strength compared to screws alone or a modified tension band technique.^[6]^ Additionally, the slight soft tissue damage and the destruction of blood supply via the percutaneous technique contributed to accelerated bone healing.

Regarding knee joint functions, the MIOT group demonstrated superior active flexion angles and Bostman functional scores, indicating better knee functions. This improvement could be linked to rigid fixation, early bone healing, and fewer complications associated with the minimally invasive technique. The VAS score at the final follow-up was significantly lower with the minimally invasive technique, owing to minimal soft tissue damage and postoperative scar formation. This aligned with the findings from previous studies.^[14,23]^ The minimal pain feeling increased the patients’ willingness to exercise their joints early, thus reducing the risk of muscle atrophy and joint adhesion.^[13]^ Furthermore, scarring from the small incision did not affect the quadriceps femoris and patellar ligament, which allowed the flexibility of the knee during flexion and extension, further improving patient’s satisfaction.

Postoperative complications in the MIOT group were notably lower, particularly in terms of delayed wound healing, owing to the avoidance of extended incisions and preservation of blood supply to the patella. Minimally invasive techniques also provided advantages in preventing skin contact with abrasions, thus reducing the risk of delayed wound healing. Implant migration, a phenomenon associated with discomfort in front of the knee joint post-operation, was exclusively observed in the ORIF group.^[24]^ A hypothesis suggested that factors such as osteoporosis and aggressive functional exercises might contribute to implant displacement.^[13]^ Delayed wound healing was another cause of implant loosening.^[14]^ Symptomatic implant irritation, more prevalent in the open surgical approach with unstable K-wire fixation,^[20]^ was less frequent in the MIOT group, possibly due to the slightly shorter lengths of screws than the initial measurement and less soft tissue disturbance. Broken wires, while not a severe complication, could interfere with normal functional rehabilitation, potentially leading to reoperation or joint stiffness. All in all, the low complication rates were directly related to minimally invasive techniques.

There were several limitations of our present study. The biggest limitation of this minimally invasive technique was that it was not suitable for those patellar fractures with multiple displaced and stepped fragments. For those comminuted fractures, precarious reduction by closed technique was expected and further research would be required. Secondly, the study lacked follow-up of late complication of joint function, such as traumatic arthritis. Finally, the retrospective nature and relatively small sample size of this study posed a risk of conclusion deviation. Larger randomized controlled trials with extended follow-ups are imperative to confirm and further elucidate these findings.

## 5. Conclusions

In conclusion, the MIOT technique emerged as both feasible and reproducible, offering satisfactory reduction with rigid fixation for transverse patellar fractures. Notably, this technique provided superior clinical outcomes, knee functions, and fewer complications compared to traditional open surgical methods. While our study contributed valuable insights, large-scale studies would be essential to comprehensively validate the effectiveness and general applicability of the MIOT technique.

## Author contributions

**Conceptualization:** Xiang-Yu Ma.

**Data curation:** Xiang-Yu Ma.

**Formal analysis:** Dong Cui.

**Funding acquisition:** Xiang-Yu Ma, Da-Peng Zhou.

**Investigation:** Dong Cui, Jia-Lin Sun, Hong Yuan, Da-Peng Zhou.

**Methodology:** Dong Cui, Jia-Lin Sun, Bing Liu.

**Project administration:** Hong Yuan.

**Software:** Jia-Lin Sun, Hong Yuan, Bing Liu, Hai-Long Yu.

**Supervision:** Jia-Lin Sun, Hong Yuan, Bing Liu, Hai-Long Yu.

**Validation:** Xiang-Yu Ma, Jia-Lin Sun, Hai-Long Yu, Tian-Yu Han.

**Visualization:** Hai-Long Yu, Tian-Yu Han.

**Writing – original draft:** Xiang-Yu Ma.

**Writing – review & editing:** Da-Peng Zhou, Hai-Long Yu, Tian-Yu Han.
